# Using transcriptomics to identify and validate novel biomarkers of human skeletal muscle cancer cachexia

**DOI:** 10.1186/gm122

**Published:** 2010-01-15

**Authors:** Nathan A Stephens, Iain J Gallagher, Olav Rooyackers, Richard J Skipworth, Ben H Tan, Troels Marstrand, James A Ross, Denis C Guttridge, Lars Lundell, Kenneth C Fearon, James A Timmons

**Affiliations:** 1 Department of Clinical and Surgical Sciences (Surgery), School of Clinical Sciences and Community Health, University of Edinburgh, EH16 4SB, UK; 2 Translational Biomedicine, Heriot-Watt University, Edinburgh, EH14 4AS, UK; 3 Department of Anaesthesiology and Intensive Care, and Department of Surgery, Karolinska University Hospital, 14186, Huddinge, Sweden; 4 Department of Biology and Biotech Research and Innovation Centre, Ole Maaloes Vej 5, University of Copenhagen, DK-2200, Denmark; 5 Division of Human Cancer Genetics, Ohio State University Medical Center, Columbus, OH 43210, USA; 6 Lifestyle Research Group, The Royal Veterinary College, 4 Royal College Street, University of London, NW1 0TU, UK; 7 Centre for Healthy Ageing, Department of Biomedical Sciences, University of Copenhagen, Blegdamsvej, DK-2200, Denmark

## Abstract

**Background:**

Cancer cachexia is a multi-organ tissue wasting syndrome that contributes to morbidity and mortality in many cancer patients. Skeletal muscle loss represents an established key feature yet there is no molecular understanding of the disease process. In fact, the postulated molecular regulators of cancer cachexia originate largely from pre-clinical models and it is unclear how these translate to the clinical environment.

**Methods:**

*Rectus abdominis *muscle biopsies were obtained from 65 upper gastrointestinal (UGI) cancer patients during open surgery and RNA profiling was performed on a subset of this cohort (n = 21) using the Affymetrix U133+2 platform. Quantitative analysis revealed a gene signature, which underwent technical validation and independent confirmation in a separate clinical cohort.

**Results:**

Quantitative significance analysis of microarrays produced an 83-gene signature that was able to identify patients with greater than 5% weight loss, while this molecular profile was unrelated to markers of systemic inflammation. Selected genes correlating with weight loss were validated using quantitative real-time PCR and independently studied as general cachexia biomarkers in diaphragm and *vastus lateralis *from a second cohort (n = 13; UGI cancer patients). CaMKIIβ correlated positively with weight loss in all muscle groups and CaMKII protein levels were elevated in *rectus abdominis*. TIE1 was also positively associated with weight loss in both *rectus abdominis *and *vastus lateralis *muscle groups while other biomarkers demonstrated tissue-specific expression patterns. Candidates selected from the pre-clinical literature, including FOXO protein and ubiquitin E3 ligases, were not related to weight loss in this human clinical study. Furthermore, promoter analysis identified that the 83 weight loss-associated genes had fewer FOXO binding sites than expected by chance.

**Conclusion:**

We were able to discover and validate new molecular biomarkers of human cancer cachexia. The exercise activated genes *CaMKIIβ *and *TIE1 *related positively to weight-loss across muscle groups, indicating that this cachexia signature is not simply due to patient inactivity. Indeed, excessive CaMKIIβ activation is a potential mechanism for reduced muscle protein synthesis. Our genomics analysis also supports the view that the available preclinical models do not accurately reflect the molecular characteristics of human muscle from cancer cachexia patients.

## Background

Cancer cachexia is a syndrome associated with malignant tumor disease defined by weight loss, asthenia and anorexia. Up to half of all cancer patients are affected, leading to increased morbidity and poor prognosis [1] with perhaps 20% of cancer deaths being related to cachexia rather than direct tumor effects [2]. Cachectic patients suffer loss of both muscle mass and adipose tissue (with comparative sparing of visceral protein) and this tissue loss appears resistant to nutritional support [3,4]. A PubMed analysis indicates that almost one-third of documents discussing cancer cachexia are review articles, highlighting the need for more primary investigations to shed light on the detailed mechanisms that produce the syndrome in patients. Furthermore, most molecular hypotheses have been generated using pre-clinical models or reflect biochemical concepts [5] and there has been little progress in relating these potential mechanisms to changes observed in the patient.

Muscle mass is maintained by physical activity, reflecting a balance between protein synthesis and degradation. Intracellular protein breakdown involves the ubiquitin proteasome pathway (UPP) and the autophagy (lysosomal), caspase, cathepsin and the calcium-dependent calpain pathways. The individual prominence of each of these pathways in muscle wasting conditions is still debated. Many of the molecular signaling pathways that are postulated to contribute to muscle atrophy in pre-clinical models mediate their effects through activation of the UPP [6]. Identification of two muscle-specific E3 ubiquitin ligases, MuRF-1 and MAFbx/atrogin-1, in a large number of animal models of atrophy [7,8] has been used to provide an argument for a major contribution of the UPP in muscle wasting, such that these genes are now measured as surrogate indicators of UPP activation. It should be kept in mind that active tissue remodeling, even with net protein accretion, may well rely partly on the protein degradation pathways and, as such, they may not represent logical surrogates for commenting on net protein degradation.

In humans, reduced levels of phosphorylated (inactive) FOXO3a have been observed in the skeletal muscle of cachectic compared with non-cachectic cancer patients, but an unexplained twofold reduction in the amount of FOXO1 and FOXO3a was also observed [9], making the data challenging to interpret. FOXO3 also appears to induce expression of autophagy-related genes [10-13], suggesting a link between the lysosomal and proteasomal systems. However, there is also evidence that the UPP is first activated with increasing weight loss then declines as the disease severity progresses [14]. This suggests that UPP is a marker of protein turn-over rather than wasting *per se *(with turn-over increasing as the muscle weakens, but only while the patient continues to be ambulatory) or that UPP proteins are not reliable biomarkers. Furthermore, recent data indicates a dissociation between protein dynamics *in vivo *and activation or expression of the UPP-related signaling molecules in human skeletal muscle [15]. Overall, it is not clear what regulates muscle mass *in vivo *nor is it clear to what extent protein degradation contributes over inhibition of protein synthesis [15,16]. Given the paucity of data derived from cancer cachexia patients, including study of the UPP and autophagy systems, we sought to carry out both targeted and global molecular profiling in the skeletal muscle of cancer patients and relate our findings to clinical status.

## Methods

Men and non-pregnant women over 18 years of age were recruited to the study from two separate centers. Written informed consent was obtained from all subjects and ethical approval received from Lothian Research Ethics Committee (UK) and the Regional Ethics Committee in Stockholm (Sweden). Participating patients had a diagnosis of upper gastrointestinal cancer (esophageal, gastric, pancreatic) and were undergoing surgery with the intent of resection of the primary tumor. A small number of weight stable (WS) patients undergoing surgery for benign, non-inflammatory conditions (n = 7) were also included in the analysis. In center 1 (Edinburgh, UK) a fasting venous blood sample was taken and serum C-reactive protein measured as a marker of systemic inflammation (SI). Body mass index (BMI) and mid-arm muscle circumference were calculated. Clinical details and degree of weight loss from self-reported pre-illness stable weight were recorded. A weight loss ≥ 5% identified weight-losing (WL) cancer patients as opposed to WS individuals. A serum C-reactive protein ≥ 5 mg/l was used to define the presence of SI. For patients from center 2 (Stockholm, Sweden) weight and self-reported change in weight over time were recorded. Rate of weight loss was therefore used in these subjects. Due to the small number of controls (otherwise considered as non-cancer patients but with other co-morbidities) and the lack of detailed knowledge of their physical capacity, the primary analysis strategy was chosen to generate molecular changes that varied with the severity of weight loss in patients in center 1 and validate such changes in the independent cohort from center 2 using more than one muscle type. This strategy was devised to provide a stringent test of the molecular changes, as the conclusions are based on a relatively large number of patients with otherwise similar clinical characteristics.

All biopsies were taken at the start of open abdominal surgery. In center 1, the edge of the *rectus abdominis *was exposed and a 1-cm^3 ^specimen removed using sharp dissection. The biopsy was snap frozen in liquid nitrogen and stored at -80°C until further analysis. In center 2, *vastus lateralis *muscle biopsies were taken with a Bergstrom needle and diaphragm biopsies were obtained by sharp dissection when possible. Both samples were snap frozen and stored at -80°C for further analysis. Approximately 20 mg of frozen tissue was homogenized in 0.5 ml of lysis buffer (Triton - X100 (1%), NaCl (150 mM), Tris-HCl (50 mM), EDTA (1 mM), PMSF (1 mM), protease inhibitors (Roche Diagnostics, Burgess Hill, UK); 1 tablet per 10 ml), water to 10 ml) using a Powergen 125 (Fisher Scientific, Loughbourgh, UK)) electric homogenizer. Samples were left on ice for 15 minutes prior to centrifuging at 13,000 rpm for 15 minutes. The supernatant was removed, and protein concentration was determined by comparing equal volumes of sample solution to known standards using the Lowry method. Samples were then stored at -80°C.

Approximately 20 mg of muscle was re-suspended in 180 μl of low salt lysis buffer (10 mM HEPES, 10 mM KCl, 1.5 mM MgCl_2_, 0.1 mM EDTA, 0.1 mM EGTA, 1 mM DTT, 0.5 mM PMSF, protease inhibitors (Roche Diagnostics; 1 tablet per 10 ml)) and ground using a handheld homogenizer. Samples were incubated on ice for 5 minutes before two cycles of freeze-thaw lysis. After a brief vortex, samples were centrifuged at 4,000 rpm for 3 minutes. The supernatant was removed and the pellet (containing the nuclei) re-suspended in 40 μl high salt extraction buffer (20 mM HEPES, 420 mM NaCl, 1 mM EDTA, 1 mM EGTA, 25% glycerol, 1 mM DTT, protease inhibitors (Roche Diagnostics; 1 tablet per 10 ml)). Samples were incubated on ice for 30 minutes with gentle mixing of the tubes every 5 to 10 minutes. Samples were centrifuged at 4,000 rpm for 5 minutes at 4°C. An aliquot of supernatant (containing the nuclear proteins) was stored at -80°C.

Protein from each sample (20 μg) was added to 3 μl of 4 × loading buffer solution (0.5 M Tris-HCl pH 6.8, 20% glycerol, 4% SDS, 0.05% β-mercaptoethanol, 0.004% bromophenol blue) and boiled for 3 minutes. Proteins were resolved using SDS-PAGE at 160 V for 45 minutes. Proteins were transferred to a nitrocellulose membrane (80 mA for 1 hour) using semi-dry transfer (Biorad, Hemel Hempstead, UK). Membranes were blocked with either 3% bovine serum albumen/tris-buffered saline (TBS) with Tween 20 (TBST; 0.05% Tween) overnight at 4°C or with 5% milk/TBST for 1 hour at room temperature. Incubation with primary antibody (1:1,000) was carried out in either 3% bovine serum albumen/TBST or 0.5% milk/TBST solution at room temperature for 2 hours or overnight at 4°C. Membranes were washed with TBST and primary antibody binding detected using horseradish-peroxidase conjugated secondary antibodies (1:2,000 to 1:5,000; anti-mouse, anti-rabbit; Upstate, Dundee, UK). Specific signal was detected using ECL reagent (GE Healthcare, Little Chalfont, UK) and exposure on photographic film (Kodak). Films were scanned and densitometry values estimated using ImageJ (NIH) software. The primary antibodies used in the study were against phos-CaMKII(Thr286), FOXO1 and FOXO3a (New England Biolabs, Hitchin, UK), Lamin A/C (Insight, Wembely, UK), alpha-skeletal actin (Novocaestra, Newcastle, UK) and calcium/calmodulin-dependent protein kinase (CaMK)II (BD Biosciences, Oxford, UK).

Total RNA was extracted from approximately 20 mg of muscle using TRIzol (Invitrogen, Paisley, UK) reagent according to the manufacturer's directions. The RNA pellet was re-suspended in diethylpyrocarbonate-treated water and RNA concentration was determined using a Nanodrop spectrophotometer (LabTech International, Ringmer, UK). RNA quality was assessed using 260/280, 230/260 ratios and the RNA integrity number (RIN) score from the BioAnalyzer 2100 instrument (Agilent Technologies, Stockport, UK). Total RNA (3.5 μg) was reverse transcribed and processed according to the protocol provided by Affymetrix Inc. for the GeneChip Expression 3' Amplification One-Cycle Target Labeling and Control Reagents kit (Affymetrix, High Wycombe, UK). Reverse transcription and second strand cDNA synthesis were followed by *in vitro *transcription and biotinylation. Biotinylated cRNA products were cleaned up using columns (Affymetrix). The quality of the biotinylated cRNA was assessed by Nanodrop (LabTech International, UK) and BioAnalyzer (Agilent Technologies) instruments and the cRNA was then fragmented according to Affymetrix protocols. Samples were hybridized to the HGU-133plus2 GeneChip array (covering approximately 54,000 sequences). The raw data files can be accessed at the Gene Expression Omnibus using the ID [GEO:GSE18832].

For quantitative real time PCR (qRT-PCR), cDNA was prepared using 1 μg RNA, TaqMan reverse transcription reagents (Applied Biosystems, Warrington, UK) and random hexamer primers (Applied Biosystems). Primers were designed to span introns using Primer Express 3.0 software (Applied Biosystems) and constructed by Invitrogen (Paisley, UK); primer sequences are detailed in Table S1 in Additional data file 1. Samples were run on an ABI 7900HT Fast Real-Time PCR system (Applied Biosystems) in triplicates of 20 μl per well using SYBR Green PCR Master Mix (Applied Biosystems) as per the manufacturer's instructions. Expression levels were normalized to ribosomal 18S RNA and results examined using the ΔCt method [17]. SPSS (SPSS Inc, Chicago, IL, USA) or GraphPad (GraphPad Software, La Jolla, CA, USA) statistical software was utilized. Student's two tailed *t*-test or one way ANOVA (analysis of variance) was used to compare means between groups. Log transformation was used when appropriate. Mann-Whitney was used for nonparametric analysis. Contingency tables were constructed where relevant and analyzed by Fisher's exact test. Statistical significance was set at *P *< 0.05.

Microarray data were analyzed using the Microarray Suite software (MAS) version 5.0 (Affymetrix). To improve the accuracy of the gene to probe relationship, a custom chip definition file (CDF) [18] was used defining the Affymetrix probes by Ensembl transcript ID. Data were normalized using MAS5 and robust multi-array average [19]. Genes called absent on every array by the MAS5 software were filtered from the data and remaining genes analyzed using the quantitative function in significance analysis of microarrays (SAM) [20] implemented in the Bioconductor suite [21]. Percentage weight loss or SI were the quantitative variables. To test the robustness of the approach, the limma package [22] in the Bioconductor suite was used to identify genes co-varying with weight loss or SI. Both SAM and limma generate a false discovery rate (FDR) [23]. All genes identified by both procedures with an FDR <10% that covaried with weight loss were further examined. We also carried out a comparative microarray analysis [24,25] to examine the link between muscle cachexia and other muscle physiological states. The top 20 most regulated genes by eccentric muscle damage [26], muscle obtained from intensive care unit patients [27] and in response to exercise training [24] were obtained from three published articles. The mean values for these highly regulated marker genes for these physiological states were then plotted using the patient values from the present study, where patients had either less than or more than 5% weight loss. Functional annotation of these genes was carried out using Gene Ontology (GO) [28] utilizing the topGO tool [29] in the Bioconductor suite along with web-based Ingenuity Pathway Analysis [30]. For analysis of microarray data the Bioconductor suite [21] and the R language for statistics (R Development Core Team; version 2.7.1) were used.

The gene-sets (see below) identified by microarray analysis were used in further investigation of the regulatory mechanisms using promoter analysis. For all genes the region up to 1,500 bp upstream of the annotated gene start was used as the proximal promoter region. Both strands were then scanned with the JASPAR [31] matrices representing various mammalian transcription factor binding sites (89 in total). A matrix specific threshold corresponding to 0.8 of the scoring range of the matrix was used on the log-ratio matrix. All log-ratio transformations were done using a zero order uniform background model and a pseudo-count of one to avoid zero-entries in the original JASPAR matrix. The number of hits per base-pair and the number of sequences with one or more hits were registered and used for over-representation statistical analysis. We used a background set of promoter sequences extracted in a similar manner from the 'all genes expressed' present/absent call in skeletal muscle from this array technology [24,27]. A sequence-specific over-representation was calculated using Fisher's exact test and a base-pair-specific over/under-representation was calculated using a Z-score. Finally, using the base-pair-specific over- and under-representation values, a heatmap was generated for visualization purposes. For all analyses the ASAP [31] framework was used in conjunction with R.

## Results

### Subject characteristics

Fifty-nine subjects were recruited over time (7 controls and 52 patients with upper gastrointestinal cancer) from center 1 (Edinburgh). Patient demographics and anthropometric characteristics are shown in Table 1. Average weight loss for center 1 cancer patients was 8.9% (range -0.5 to 43.8%). Compared to the control group, cancer patients had significant weight loss (*P *< 0.001) and had a lower BMI (*P *= 0.001). The controls were substantially younger (*P *= 0.009) and hence could not be used as a case-control comparison group for the molecular profiling. Instead, gene expression was related to body mass status. WL cancer patients had a lower BMI (*P *= 0.010) than WS cancer patients. The Affymetrix GeneChip studies used a subset of 21 patients from the cohort in center 1, where high quality RNA was available at the time of gene-chip analysis (Table 2). BMI and mid-arm muscle circumference were not significantly different between the 'Affymetrix cohort' and the larger group of cancer patients. To validate the findings in the first group of patients ('Affymetrix cohort') a second group of 13 patients with esophageal cancer was recruited from an independent clinical center (center 2, Sweden). Patients of this group were similar to the cancer patients from center 1 (Table 1).

**Table 1:** Clinical data for patients and control subjects from centers 1 and 2

	Center 1	Center 1	Center 2
	no-cancer	patients	patients
	(n = 7)	(n = 52)	(n = 13)
Male/female	5/2	34/18	12/1
Age (years)	51 (5.5)	66 (1.3)*	65 (1.5)*
% weight loss	0	8.9 (1.1)*	7.7 (2.0)*
BMI	30.6 (1.3)	25.5 (0.5)*	25.5 (1.2)
CRP	2.8 (0.7)	17.4 (4.4)	-
MAMC	25.9 (1.3)	24.4 (0.4)	-

Mean (standard error of the mean) values are presented. *P < 0.05 compared with center 1 control. Center 1: Edinburgh, UK; centre 2: Stockholm, Sweden. BMI: body mass index; CRP: C reactive protein; MAMC: mid-arm muscle circumference.

### Microarray analysis: novel genes associated with weight loss in cancer (centre 1)

The microarray study was undertaken on *rectus abdominis *muscle from a subgroup of center 1 patients (Table 2). Hierarchical and k-means clustering were undertaken with normalized data, using a gene list where those with a low standard deviation were removed. No pattern emerged from this analysis. Using the probe-sets that detect atrogenes (genes reproducibly detected in pre-clinical models of cachexia), which we have previously demonstrated reliably change in human skeletal muscle sepsis [27], we carried out hierarchical and k-means clustering. No pattern emerged from this analysis. Thus, our first attempted analysis did not yield any data in support of pre-clinical studies [32] and also demonstrated that muscle cancer cachexia appears distinct from the inflammation-driven skeletal muscle remodeling observed in the intensive care unit [27].

**Table 2:** Demographics of controls and cancer patients included in the Affymetrix analysis from centre 1

	No-cancer	Cancer patients	
	(n = 3)	(n = 18)	*P*
Male/female	2/1	12/6	-
Age (years)	45(2)	67(2)	<0.001
% weight loss	0	8.9(1.6)	<0.001
BMI	28.5(1.7)	24.4(0.8)	0.080
CRP	2.7(0.9)	19.7(8.1)	0.052
MAMC	23.8(1.7)	23.7(0.5)	0.960

Mean (standard error of the mean). BMI: body mass index; CRP: C reactive protein; MAMC: mid arm muscle circumference.

We then identified genes that varied with percentage weight loss using the quantitative SAM methodology [20]. In this multiple comparison corrected correlation analysis, the WS group contained both cancer patients and three non-cancer controls in order to identify *bona fide *cachexia associating genes. SAM identified 74 genes with a FDR between 0 and 10% (most <5% FDR) that covaried positively with weight loss, and nine genes with a FDR between 0 and 10% (most <5% FDR) that covaried negatively with weight loss (Additional data file 2). Correlation coefficients (R) for these 83 genes were generated using Pearson's product moment correlation. Positive coefficients ranged from 0.82 to 0.57 (*P *< 0.01), and for negatively correlating genes, R ranged from -0.74 to -0.65 (*P *< 0.01). Each relationship was visually inspected by plotting the data.

**Figure 1:**
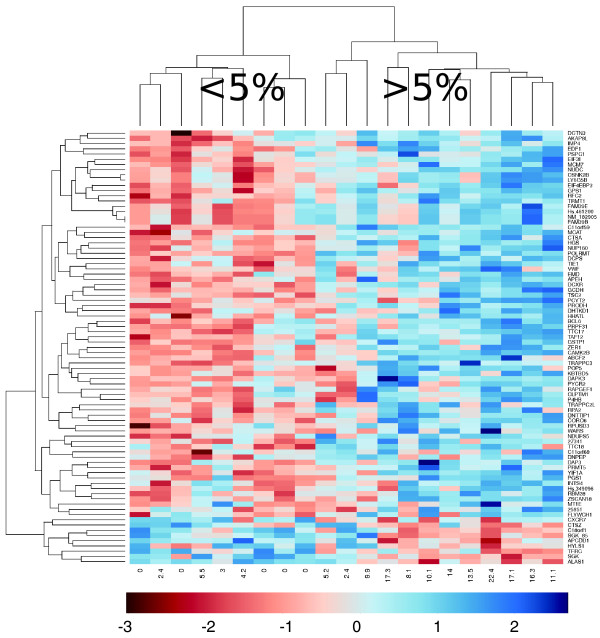
**Cluster analysis identifies high and low weight loss groups**. Using SAM and limma, 83 genes were identified as correlating with weight loss. Expression data from these genes were used to drive cluster analysis. This revealed two clusters of subjects; high weight loss (≥ 5%) and low weight loss (<5%).

**Figure 2:**
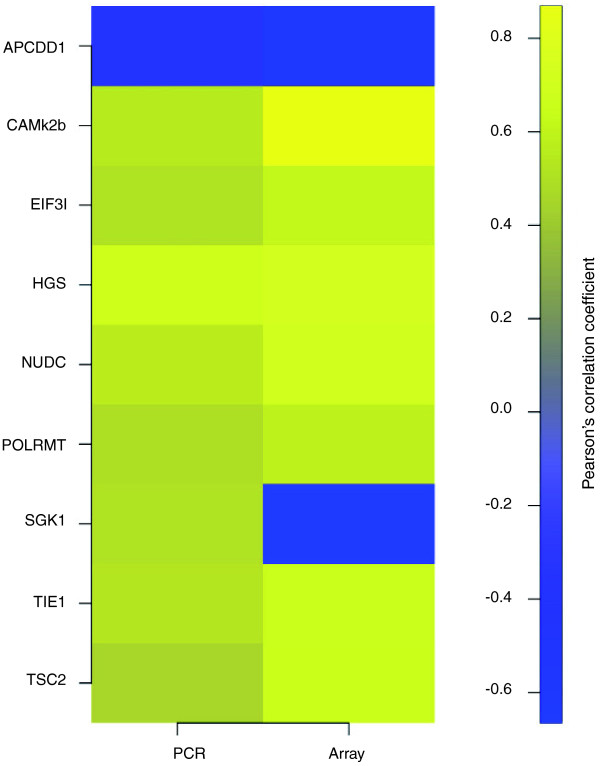
**qRT-PCR validates array-identified genes covarying with weight loss**. For each of the genes validated by qRT-PCR Pearson correlation coefficients were generated for expression and percentage weight loss for both the Affymetrix data and the qRT-PCR data. All genes except *SGK1 *validated the array data. *P*-values for the correlations ranged from 0.03 to below 0.01. Yellow indicates positive correlation; blue indicates negative correlation.

Most of the genes correlating with weight loss had not been associated previously with cachexia in humans or animal models. Notably, FOXO transcription factors and the E3 ligases MURF1 and MAFbx were absent from this list. Simple cluster analysis revealed visual distinction of patients with <5% reported weight loss from those with >5% reported weight loss (Figure 1). This Affymetrix-derived WL gene signature was technically validated by qRT-PCR of the 9 genes (*APCDD1*, *CaMKIIβ*, *EIF3I*, *HGS*, *NUDC*, *POLRMT*, *SGK*, *TIE1 *and *TSC2*). Eight validated the microarray data, with only *SGK *expression being inconsistent with the Affymetrix analysis (Table 3 and Figure 2; Supplemental figure 1 in Additional data file 3).

**Table 3:** Genes correlating with weight loss

	Center 1 (n = 21)			Center 2 (n = 13)			
		
	Gene-chip			RT-qPCR			
		
Gene	CC *rectus abdominis*	CC *rectus abdominis*	Regression *P*-value	CC *vastus lateralis*	Regression *P*-value	CC diaphragm	Regression *P*-value
*APCDD1*	-0.74	-0.51	0.03	0.26	NS	-0.20	NS
***CAMk2B***	**0.82**	**0.50**	**0.01**	**0.45**	**0.06**	**0.50**	**0.03**
*EIF3I*	0.64	0.50	0.02	0.10	NS	0.20	NS
*HGS*	0.7	0.67	0.00	0.17	NS	0.20	NS
*NUDC*	0.65	0.72	0.00	0.13	NS	0.0	NS
*POLRMT*	0.6	0.51	0.02	0.07	NS	0.0	NS
***TIE1***	**0.67**	**0.53**	**0.01**	**0.70**	**0.003**	**0.0**	**NS**
*TSC2*	0.69	0.47	0.03	0.40	0.1	0.0	NS

Significance analysis of microarrays (SAM) identified 82 genes correlating with weight loss. qRT-PCR validated eight of nine selected targets from this list (correlation coefficient (CC)). These eight genes were also examined in the cohort from center 2 using RNA extracted from anatomically distinct regions. For each gene the correlation coefficient from the Affymetrix data set is shown followed by the correlation coefficient for qRT-PCR and a *P*-value for this latter regression. NS: not significant.

### Candidate gene approach: analysis of FOXO transcription factors and components of the ubiquitin proteasome and autophagy pathways (centre 1)

While the microarray analysis did not yield any evidence for proteolytic pathways being upregulated, as seen in intensive care unit patients with the same gene chip technology [27], investigation of components of these pathways was nevertheless undertaken in parallel to the gene-chip study. There was no difference in the nuclear level of FOXO1 and FOXO3a protein by western blotting when patients were grouped according to weight loss. Expression of the E3 ligases MURF1 and MAFbx was examined by qRT-PCR and no relationship between mRNA expression and weight loss was found (data not shown). The autophagy-related genes *GABRAPL1 *and *BNIP3 *were modestly increased in WL patients compared to WS patients or controls (fold change = 1.46 versus 1.23 versus 1.07, respectively; *P *= 0.047). However, this *P*-value is unadjusted for the previous array analysis and may not be reliable. Both genes demonstrated a positive association with systemic inflammation (Table S2 in Additional data file 1 and Figure S2 in Additional data file 3).

### Confirmation of genes associated with weight loss in cancer cachexia (center 2)

**Figure 3:**
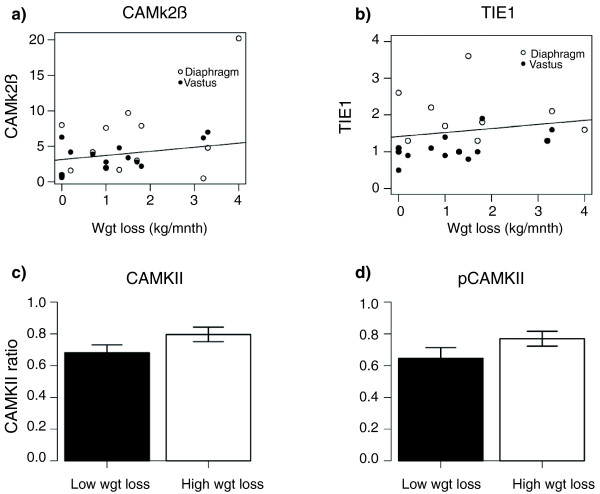
**CAMkIIβ and TIE1 correlate with weight loss in cancer cachexia**. In order to validate the findings from the *rectus abdominis*, qRT-PCR was used to examine mRNA expression of **(a) **CAMkIIβ and **(b) **TIE1 in diaphragm (open circles) and *vastus lateralis *(closed circles) in a separate clinical cohort. Correlation plots for mRNA level against rate of weight loss are shown. Correlation coefficients were significant with *P *< 0.05. CAMkII protein and phospho-protein levels are increased in subjects with weight loss. **(c) **Protein levels of CAMkII and **(d) **phosphoCAMkII were assessed in the *rectus abdominis *muscle from center 1 subjects by western blot. Intensity levels were normalized against alpha-skeletal actin and the mean ratio of CAMkII/actin or phosphoCAMkII (pCAMkII)/actin are shown for subjects with less than (black) or more than (white) 5% weight loss. *P-value < 0.05, one-sided Mann Whitney test; n = 59. Error bars represent SEM.

To validate the WL gene signature generated in *rectus abdominis *muscle from the center 1 cohort, nine genes were profiled using qRT-PCR (*APCDD1*, *CaMKIIβ*, *EIF3I*, *HGS*, *NUDC*, *SKG*, *POLRMT*, *TIE1 *and *TSC2*) in two additional types of skeletal muscle obtained from cancer cachexia patients. The significant association between CaMKIIβ and weight loss observed in *rectus abdominis *muscle from center 1 (R = 0.82, *P *= 0.01; Table 1) was reproduced (Figure 3a) in both *vastus lateralis *(R = 0.45, *P *= 0.06) and diaphragm muscle (R = 0.5; *P *= 0.03) from center 2 patients. In addition, *TIE1*, which significantly correlated with weight loss in *rectus abdominis *(R = 0.67, *P *= 0.01; Table 1) demonstrated a similar (Figure 3b) relationship in *vastus lateralis *(R = 0.7, *P *= 0.003) but not in diaphragm. Given the changes observed for *CaMKIIβ *mRNA, the protein and phosphorylation level of CaMKII in all of the *rectus abdominis *muscle obtained in center 1 was evaluated. Material from a total of 59 patients was available at the time the analysis was carried out (recruitment was ongoing beyond the time the microarray was carried out). Western blotting for both CaMKII (Figure 3c) and phosphorylated CaMKII (Figure 3d) revealed a small but significant (*P *= 0.04 and 0.07, respectively) increase in WL patients compared with the expression determined in WS patients and controls.

### Gene interaction and promoter analysis

In order to generate valid pathway or ontological enrichment scores, it is essential to relate the modulated gene list with the genes detectably expressed in the tissue of interest and not with the genome as a whole (or the entire gene-chip content). The nature of the 83-gene WL gene signature was explored in detail using GO. The highest ranked GO biological process activity from the DAVID webtool [33] was proline metabolism (*P *= 0.03). This was confirmed with the topGO [29] and GOStats [34] tools in Bioconductor. Proline metabolism has a role in collagen formation and increased collagen deposition has been noted in the muscle of cachectic cardiac failure patients [35]. Network analysis using Ingenuity [30] revealed several interactions that involve the 83 WL genes, including a calmodulin kinase gene network (Figure S3A in Additional data file 3), supporting the wet-lab data and indicating that CaMKIIβ activation appears to be a general marker of muscle wasting in human cancer cachexia. A second illustrative pathway (Figure S3B in Additional data file 3) features GLUT-4 (glucose transporter type 4) and interleukin-6, both of which are implicated in skeletal muscle metabolism [36]. This network also forms numerous connections with the glucocorticoid and androgen receptors, which may be involved in regulating skeletal muscle mass. It should be noted that despite using a back-ground gene expression file in Ingenuity [30] for genes only detected as being expressed in human skeletal muscle (approximately 21,000 probe sets, based on MAS5 present-marginal calls) the Ingenuity network analysis still included genes that may not be robustly expressed and should be used in a qualitative hypothesis generation manner.

**Figure 4:**
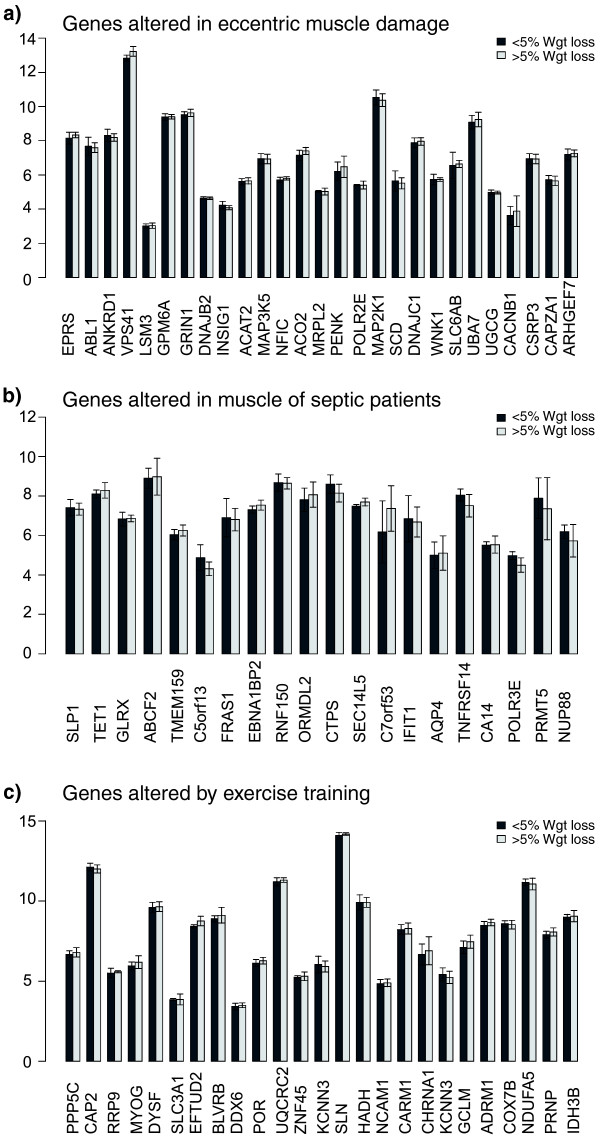
**Gene expression signatures demonstrate lack of relationship between weight loss and muscle damage, muscle sepsis and exercise training status**. The top 20 most regulated genes by **(a) **eccentric muscle damage, **(b) **muscle obtained from intensive care unit patients and **(c) **in response to exercise training were obtained from three published articles (see Methods). The mean values for these selected genes were then plotted for patients in the present study that had either less than or more than 5% weight loss. As can be observed, no single gene, for each of these 'comparative' conditions, was differentially expressed; thus, the gene expression profile of cancer cachexia does not resemble muscle damage, sepsis-induced degeneration or exercise training status. Error bars represent SEM.

Gene sequence analysis of the WL gene-set was carried out to provide insight into the potential coordinators of this expression signature. Interestingly, FOXO transcription binding sites tended to be, if anything, significantly under-represented in the human cachexia WL gene set, supporting the wet-lab analysis. Binding sites for SP1, ARNT.AHR (the hypoxia signaling partner) and TFAP2A (Transcription factor AP2-alpha or AP2) in particular, were over-represented in the proximal promoters of the WL-associated genes (Figure S4 in Additional data file 3). The analysis further supports the idea that this list is distinct. Interestingly, the enriched TF binding sites may function as clock genes, controlling circadian rhythm [37]. Another strategy for generating hypotheses for factors that might regulate a set of genes is to carry out comparative expression analysis [25], where two physiological studies are contrasted using global gene chip data. In this case we present data that patients with greater weight loss do not appear to have a common overlap with muscle damage, muscle degeneration in sepsis or muscle remodeling in exercise training (Figure 4).

## Discussion

Cancer cachexia is thought to arise due to an imbalance of the anabolic and catabolic pathways partly driven by pro-inflammatory cytokines with consequent loss of muscle mass (along with an accompanying loss of adipose tissue). In the present study, the expression of 74 genes correlated positively with weight loss in cancer cachexia subjects and that of 9 correlated negatively with it. Validation of these genes by qRT-PCR provided excellent technical confirmation of the microarray results. Biological validation of TIE1 and CaMKIIβ expression in an independent clinical cohort across distinct muscle groups, along with supportive network analysis, provides weight to the claim that these are useful markers of cancer cachexia in humans. Contrary to evidence from animal models [7,8,11], there were no significant differences in expression of the E3 ligases MURF1 and MAFbx, while FOXO protein activity was unchanged in WL compared to WS patients. These observations, combined with the array and promoter analysis, make it seem unlikely that FOXO transcription factors regulate the molecular signature of cachexia in human skeletal muscle, challenging the relevance of the pre-clinical literature in this field.

### Novel human cancer cachexia markers

The significant correlation of *CaMKIIβ *mRNA expression with weight loss along with the small but significant change in protein levels in *rectus abdominis *suggests that CaMKIIβ could be directly involved in human cancer cachexia. *CaMKIIβ *mRNA also increased with weight loss in *vastus lateralis *and diaphragm. The serine/threonine kinase CaMKII holoenzyme is activated by Ca^2+^/calmodulin, leading to autophosphorylation and maintenance of CaMKII activity even after the Ca^2+ ^signal has diminished [38]. CaMKIIβ is expressed in skeletal muscle, and levels of the protein as well as its phosphorylation status and activity increase after exercise training [39]. The relationship between CaMKIIβ expression and cachexia observed in the present study implies that the cancer cachexia profile is not simply 'physical inactivity'. In addition, it has recently been demonstrated that Ca(2+)-CaM-eEF2K signaling may be responsible for acute exercise-induced inhibition of muscle protein synthesis [40] and it is thus conceivable that chronic inappropriate activation of this 'endurance training'-related signaling molecule [24] subdues normal maintenance of skeletal muscle mass. Additional factors that could modulate CaMKII activity include alterations in lipid metabolism [41].

The significant positive correlation for *TIE1 *mRNA expression with weight loss in both the *rectus abdominis *and *vastus lateralis *muscle groups supports the idea that some chronic training-related genes are up-regulated in cachexia. In animal models TIE1 is required for normal vascular network development [42] while increased *TIE1 *mRNA levels in human skeletal muscle in response to physiological adaptation to exercise training has been demonstrated [43]. Whilst the ligands and signaling pathways of TIE1 are poorly understood, this receptor can interact with phosphoinositide 3-kinase and lead to phosphorylation and activation of Akt, protecting cells from apoptosis [44]. In functional terms, the up-regulation of TIE1 may therefore represent a protective mechanism to oppose apoptosis of some components of skeletal muscle tissue, for example, the vascular endothelium. TIE1 has also recently been linked to an *in vitro *endothelial inflammatory response [45] while an inflammatory gene signature has been shown to develop throughout surgical procedures in muscle [46]; thus, it could be argued that some component of our gene signature may be related to surgery. However, all biopsies were taken at the earliest point in surgery after the initial incision.

Furthermore, the correlation of TIE1 expression with weight loss and the lack of any further appreciable inflammatory signature would argue against this possibility. In addition, there was no evidence that the muscle profile was that of damage or that observed with systemic inflammation associated with multiple organ failure (Figure 4). It is also notable that (other than *TIE1*, *CaMKII*, *CTSA *and *PRODH*) the WL gene signature does not share similarities with the approximately 500-gene endurance exercise training gene signature [24], suggesting that the reason for elevated TIE1 and CaMKIIβ remains to be determined. It may be inappropriate partial muscle activity signaling but clearly is not simply increased muscle usage (however unlikely that might have seemed in such patients). However, the increased *CaMKIIβ *mRNA levels associated with weight loss across a range of muscle tissues imply that these muscle groups develop dysregulation of calcium sensing or are burdened by greater loading in the face of failing muscle function connected with, for example, loss of contractile machinery or impaired energy metabolism [47].

Finally, recent work has clarified two potential calcium-independent activation pathways for CaMKII. Generation of reactive oxygen intermediates can increase or prolong CaMKII activity, perhaps through inhibition of protein phosphotases that normally limit CaMKII activation [48]. CaMKII has also been implicated in muscle adaptation through phosphorylation of HDAC5 leading to MyoD/MEF2-driven differentiation of muscle cells [49]. It is plausible, therefore, that CaMKII activation is a compensatory strategy in the face of failing protein synthesis. Alternatively, the CaMKIIβ response may indicate failure of calcium homeostasis, a factor that would also activate proteolytic activities such as calpains and caspases [50,51]. It is thus possible that CaMKIIβ activation occurs at an early stage of cachexia in humans, providing an early 'read-out' on altered calcium handling.

### Human versus animal-model cancer cachexia markers and study limitations

Given the robust increase in expression of the E3 ligases reported previously in various animal models of cachexia [7,8,32], it is surprising that neither microarray nor qRT-PCR detected any regulation of MuRF1 and MAFbx. Furthermore, the 83-gene WL gene signature bore no resemblance to the Atrogene gene expression signature [27,32] generated using gene-chips. This is not due to gene-chip technology being unable to establish parallels between animal models and humans, as it has previously been demonstrated that gene expression in skeletal muscle of intensive care unit patients resembles, in part, that found in these animal models [27,32]. Indeed, results of E3 ligase expression analysis from other human models of cachexia have been contradictory. Studies including patients following bed rest, amputation for vascular disease, limb immobilization, chronic obstructive pulmonary disease, amyotrophic lateral sclerosis and ageing have demonstrated both increased and decreased expression of MuRF1 and MAFBx [52-56]. This would suggest that the ubiquitin E3 ligases do not play the same role in human cancer cachexia as that previously demonstrated in animal and cell studies. In lung cancer patients with mean weight loss of 2.9%, there was no evidence of UPP activation [57] while other human studies in patients with gastric cancer and mean weight losses of 5.2% and 5.6% have shown increases in components of the UPP [58,59]. In the present study we could not find any support for this finding, despite similar degrees of cachexia. However, cancer cachexia encompasses a spectrum progressing from early weight loss through to severe muscle wasting. The prominence of the individual proteolytic pathways at different time points along this spectrum is yet to be determined and one must keep in mind that during severe tissue wasting, both breakdown (and of course synthesis) may well be reduced with the net balance between the two widened.

A role for autophagy in human cancer cachexia has not been investigated extensively. Increased cathepsin D and acid phosphatase activity has been demonstrated in patients with varying tumor types and degrees of weight loss, suggesting that increased lysosomal activity may contribute to the development of cachexia [60]. More recently, lung cancer patients undergoing resection were shown to have increased levels of cathepsin B mRNA in skeletal muscle compared with controls [57]. The analyses examined GABARAPL1 and BNIP3. GABARAPL1 is an Atg8 homologue important in the formation of the autophagosome [61] and BNIP3 has been found to play a predominant role in induction of autophagy in rodent skeletal muscle [11]. Autophagy can be induced by starvation of amino acids, which may explain the modest increase in BNIP3 and GABARAPL1 in patients with SI where the acute phase response is activated (mobilizing amino acid from muscle to liver for consumption) and where food intake may be reduced due to anorexia or dysphagia. However, no relationship was found between these genes and patient weight loss.

A limitation of the current study is that we focus on changes in total body mass and this does not tell us about the relative contributions from lean body mass and adipose tissue. Our muscle gene expression clustering results indicate, however, that there is a skeletal muscle molecular signature that reflects changes in whole body mass and it is hard to conceive that this is not somehow reflecting the changes in the muscle tissue. A further consideration is adequate control for confounding parameters, such as inflammation, damage and physical activity. While these are difficult to directly control, we produced an analysis to suggest that such processes were unrelated to our new human muscle cancer cachexia signature (Figure 4).

## Conclusions

Human cancer cachexia is a chronic process and weight loss is not as rapid and generally not as severe as the acute muscle wasting observed in animal models. Thus, the physiological regulators are most likely very distinct in each scenario. We found increased expression of two 'endurance exercise'-activated genes, *CaMKIIβ *and *TIE1*, across different muscle groups in human cancer cachexia. Whether these could contribute to a reduction in protein synthesis remains to be ascertained.

## Abbreviations

BMI: body mass index; CaMK: calcium/calmodulin-dependent protein kinase; DTT: dithioreitol; FDR: false discovery rate; GO: Gene Ontology; MAS 5.0: Microarray Suite; PMSF: phenylmethanesulfonyl fluoride; qRT-PCR: quantitative reverse transcriptase PCR; SI: systemic inflammation; SAM: significance analysis of microarrays; TBS: tris-buffered saline; TBST: TBS with Tween 20; UPP: ubiquitin proteosome pathway; WL: weight losing; WS: weight stable.

## Competing interests

This project was assisted in part by an Affymetrix Translational Medicine award (JT) that reduced the cost of the gene-chip analysis. Affymetrix were not involved in any aspect of the data analysis or interpretation and did not influence the manuscript in any way. The authors declare that they have no competing interests.

## Authors' contributions

The genomics analysis strategy and statistical analysis was developed and carried out by JAT and IJG. Wet-lab genomic analysis was carried out by IJG, NAS, TM, OR and JAT. Western analysis was carried out by NAS, DCG and JAR. The manuscript was drafted by JAT and IJG. The manuscript was edited by IJG, NAS, JAT, TM, OR, JAR, DCG and KCHF. The clinical biobank materials were established by RJES, KCHF, NAS, LL, OR and BT. All authors have given final approval to the article.

## Additional data files

The following additional data are available with the online version of this paper: a Microsoft word file detailing primers used in the study, genes associated with systemic inflammation and data on autophagy pathway genes (Additional data file 1); an Excel spreadsheet of genes associated with weight loss or systemic inflammation in cancer cachexia (Additional data file 2); a Powerpoint file with figures and figure legends for supplementary figures referred to in the text (Additional data file 3).

## Supplementary Material

Additional file 1 Primers used in the study, genes associated with systemic inflammation and data on autophagy pathway genes. Click here for file

Additional file 2 Genes associated with weight loss or systemic inflammation in cancer cachexia. Click here for file

Additional file 3 Figures and figure legends for supplementary figures referred to in the text. Click here for file
