# Supplementary material for: Using transcriptomics to identify and validate novel biomarkers of human skeletal muscle cancer cachexia
Source: Genome Med. 2010 Jan 15;2(1):1. doi: 10.1186/gm122 (PMC2829926; doi:10.1186/gm122)
Supplement: Additional file 1 — Primers used in the study, genes associated with systemic inflammation and data on autophagy pathway genes. [file gm122-S1.doc]

### Table S1 – Primer Sequences

| **Gene Name** | **Forward Sequence** | **Reverse Sequence** |
| --- | --- | --- |
| ***APCDD1*** | GCA CCG AGT TCG TGT TCA A | TCC CGT TGA AGA CGT TGA G |
| ***BNIP3*** | GTC AAG TCG GCC GGA AAA TA | GCG CTT CGG GTG TTT AAA GA |
| ***CaMK2β*** | GAC GGC AAG TGG CAG AAC | CAA CTC TGT CCG GCG AAA |
| ***EIF3I*** | GAA GCC ATG GAT GTA ACC ACA | AGG TCC AAA GTG ACC CTT GA |
| ***GABARAPL1*** | CCA CCG CAA GGA GAC AGA AG | GAA AAT GTG ATG ACG GTG TGT GT |
| ***HGS*** | ACA GAC TCT CAG CCC ATT CC | AGA CTC GCC ATT GTG GAA CT |
| ***MAFBX*** | CCG GCT GTT GGA GCT GAT A | TTG GGC GAT GCC ACT CA |
| ***MURF1*** | GCT AGG CGT GGC TCT CAT TC | TCC TGG ATC AGG CTC GAC TT |
| ***NUDC*** | AAG AAC GGC AGC CTT GAC T | GTC CTT CTC ATC TTC CTC CTC A |
| ***POLRMT*** | AGA CCA AGA CCG CAG GAA G | CTC CGA CAC GCT CTC AGC |
| ***SGK1*** | TTT CCA AAG AGG GGT TCT CC | TGG CAT GAT TAC ATG GCT CT |
| ***TIE1*** | GCC CAG ATT GCG CTA CAG | ATC AAT GCC CGC GTA AGT |
| ***TSC2*** | GCA GCA TCA GTG TGT CTG AAC | AAG CTG GCA CTG GTG AGG |

### Microarray analysis: novel genes associated with SI in cancer (Centre 1)

Using plasma CRP concentration as an index of SI, a provisional analysis of the microarray data was performed as described above for weight loss. This analysis was not corrected for further multiple testing beyond that afforded by the FDR calculation in SAM. Patients with no SI (CRP <5mg/l) were included to identify inflammation related genes rather than genes associated with cancer alone. 76 genes (FDR<10%) positively correlated with SI and 25 genes (FDR<10%) had a significant negative correlation (**Additional file 3)**. Correlation coefficients ranged from 0.82 to 0.44 for positively correlating genes and -0.44 to -0.72 for negatively correlating genes (p<0.05). Positively correlating genes included the transcription factor *FOXO1* and the autophagy related gene *GABARAPL1* but not *MURF1* and *MAFbx*. In contrast to the WL genes, clustering with this gene-list did not easily differentiate high from low SI subjects and there was no overlap between SI genes and WL genes. Given these findings and the lack of correction for additional multiple comparisons this analysis should be considered speculative.

### Table S2 – Expression of autophagy genes associated with weight loss or systemic inflammation in cancer patients from Centre 1

**Control Cancer patients**

**WS WL p no SI SI present p**

(n=7) (n=20) (n=32) (n=26) (n=26)

**BNIP3** 1.07(0.57) 1.23(0.44) 1.46(0.50) 0.047 1.20(0.48) 1.54(0.43) 0.003

**Gabarapl1** 1.10(0.57) 1.74(0.95) 1.52(0.61) 0.063 1.45(0.66) 1.75(0.82) 0.018

**Notes:** Mean (SEM) values are presented. P values are unadjusted.
