# Supplementary material for: Using transcriptomics to identify and validate novel biomarkers of human skeletal muscle cancer cachexia
Source: Genome Med. 2010 Jan 15;2(1):1. doi: 10.1186/gm122 (PMC2829926; doi:10.1186/gm122)
Supplement: Additional file 3 — Figures and figure legends for supplementary figures referred to in the text. [file gm122-S3.ppt]

## Slide 1
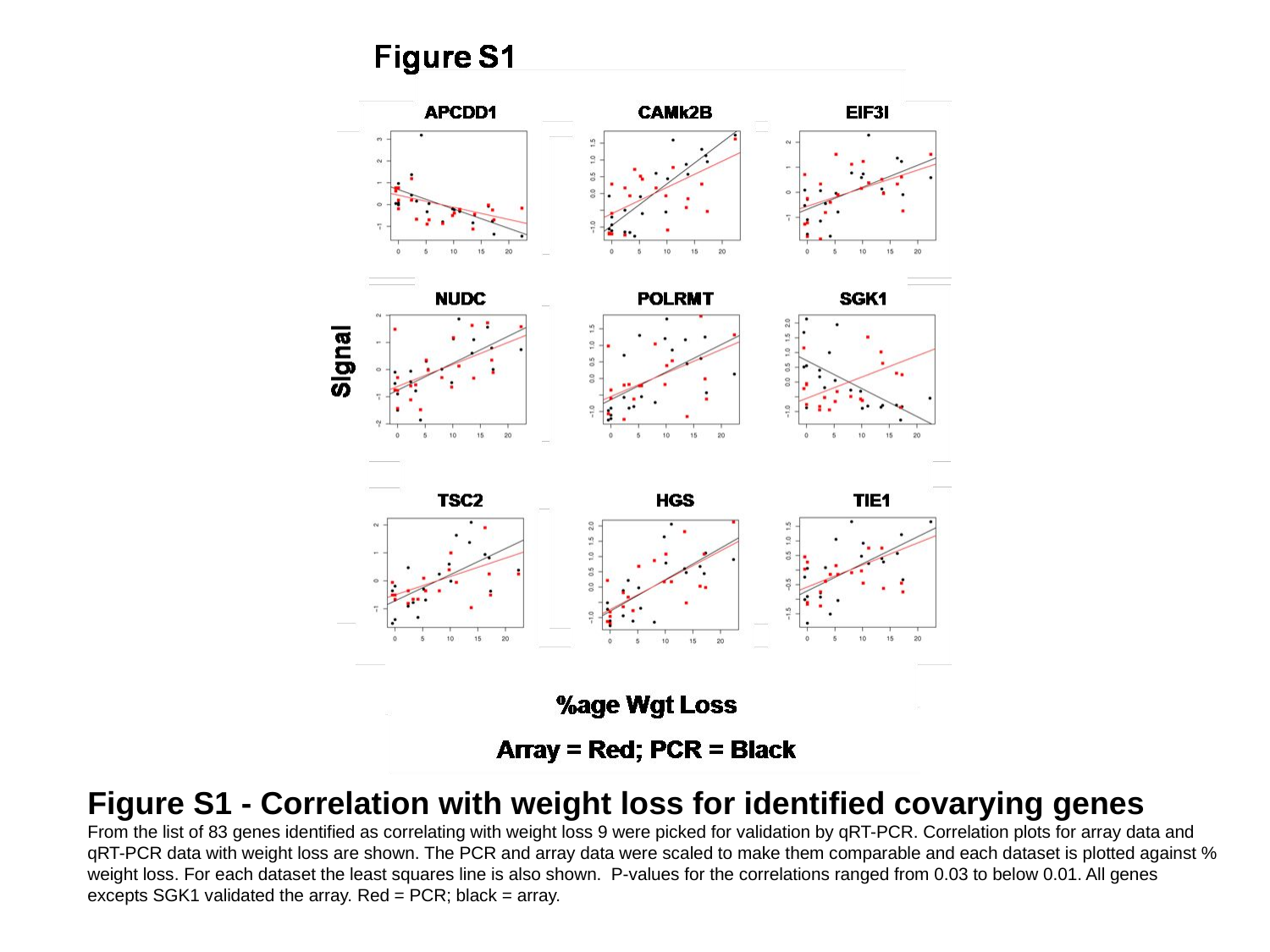

Figure S1 - Correlation with weight loss for identified covarying genes
From the list of 83 genes identified as correlating with weight loss 9 were picked for validation by qRT-PCR. Correlation plots for array data and qRT-PCR data with weight loss are shown. The PCR and array data were scaled to make them comparable and each dataset is plotted against % weight loss. For each dataset the least squares line is also shown. P-values for the correlations ranged from 0.03 to below 0.01. All genes excepts SGK1 validated the array. Red = PCR; black = array.

## Slide 2
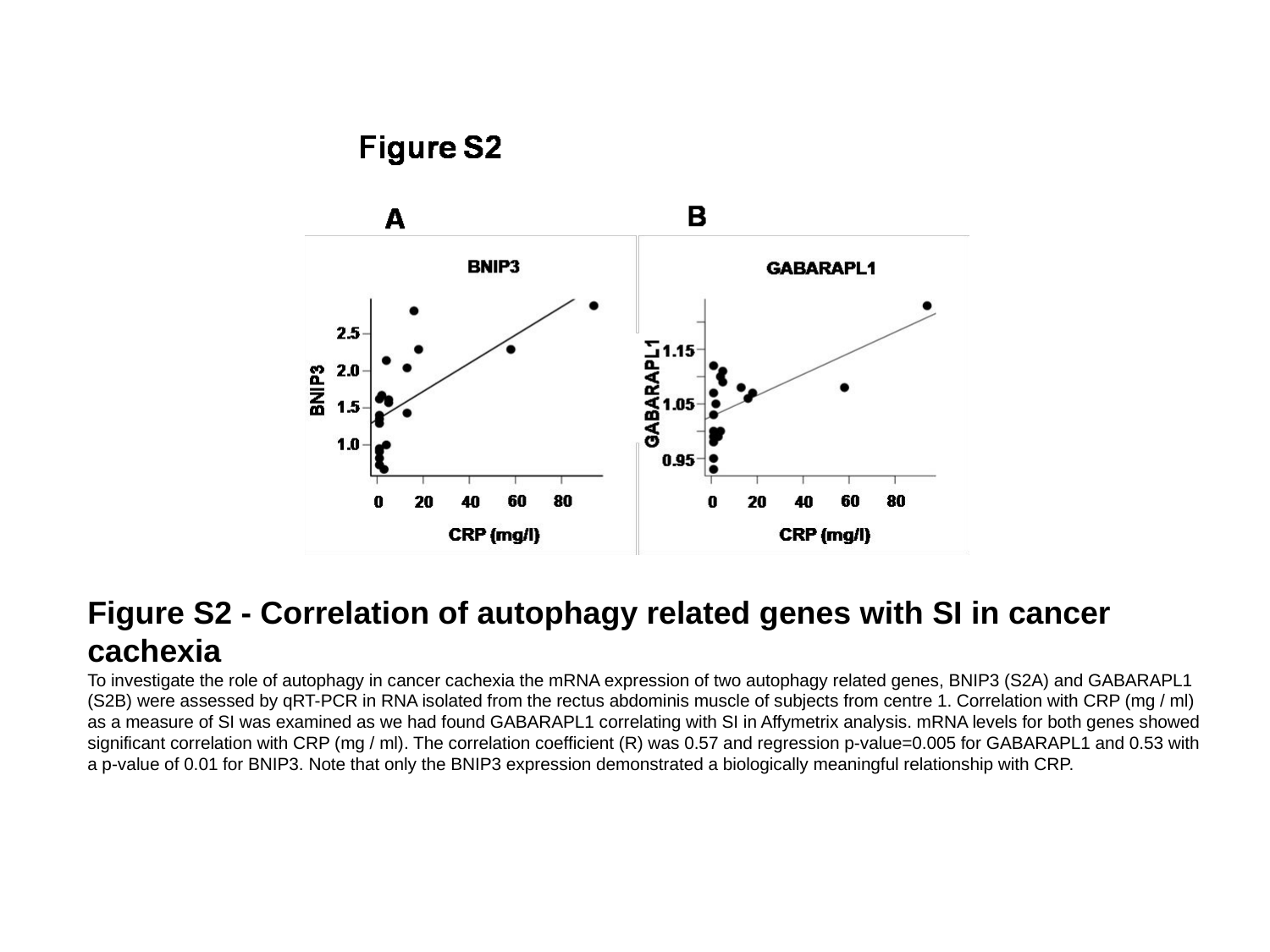

Figure S2 - Correlation of autophagy related genes with SI in cancer cachexia
To investigate the role of autophagy in cancer cachexia the mRNA expression of two autophagy related genes, BNIP3 (S2A) and GABARAPL1 (S2B) were assessed by qRT-PCR in RNA isolated from the rectus abdominis muscle of subjects from centre 1. Correlation with CRP (mg / ml) as a measure of SI was examined as we had found GABARAPL1 correlating with SI in Affymetrix analysis. mRNA levels for both genes showed significant correlation with CRP (mg / ml). The correlation coefficient (R) was 0.57 and regression p-value=0.005 for GABARAPL1 and 0.53 with a p-value of 0.01 for BNIP3. Note that only the BNIP3 expression demonstrated a biologically meaningful relationship with CRP.

## Slide 3
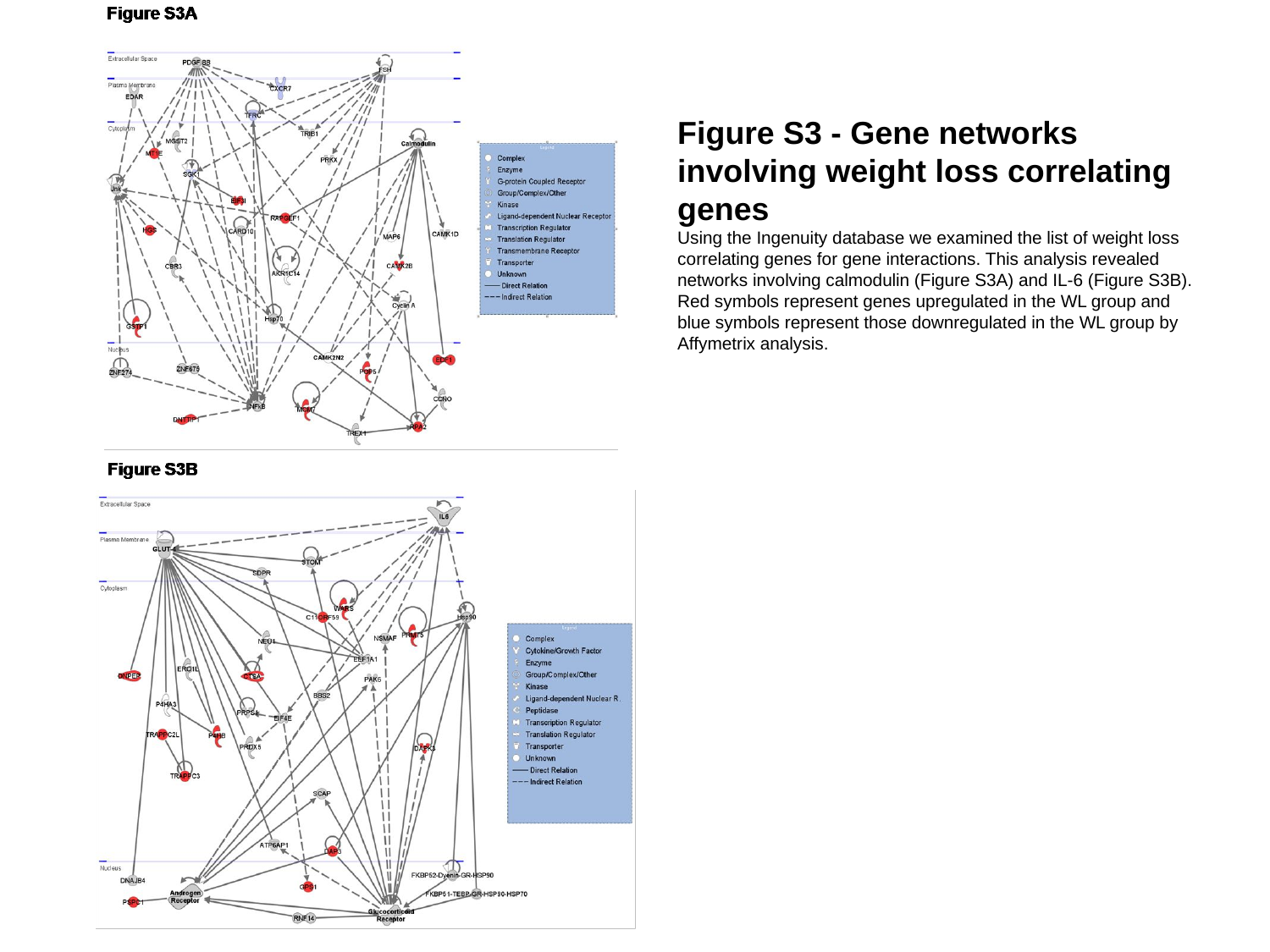

Figure S3 - Gene networks involving weight loss correlating genes
Using the Ingenuity database we examined the list of weight loss correlating genes for gene interactions. This analysis revealed networks involving calmodulin (Figure S3A) and IL-6 (Figure S3B). Red symbols represent genes upregulated in the WL group and blue symbols represent those downregulated in the WL group by Affymetrix analysis.

## Slide 4
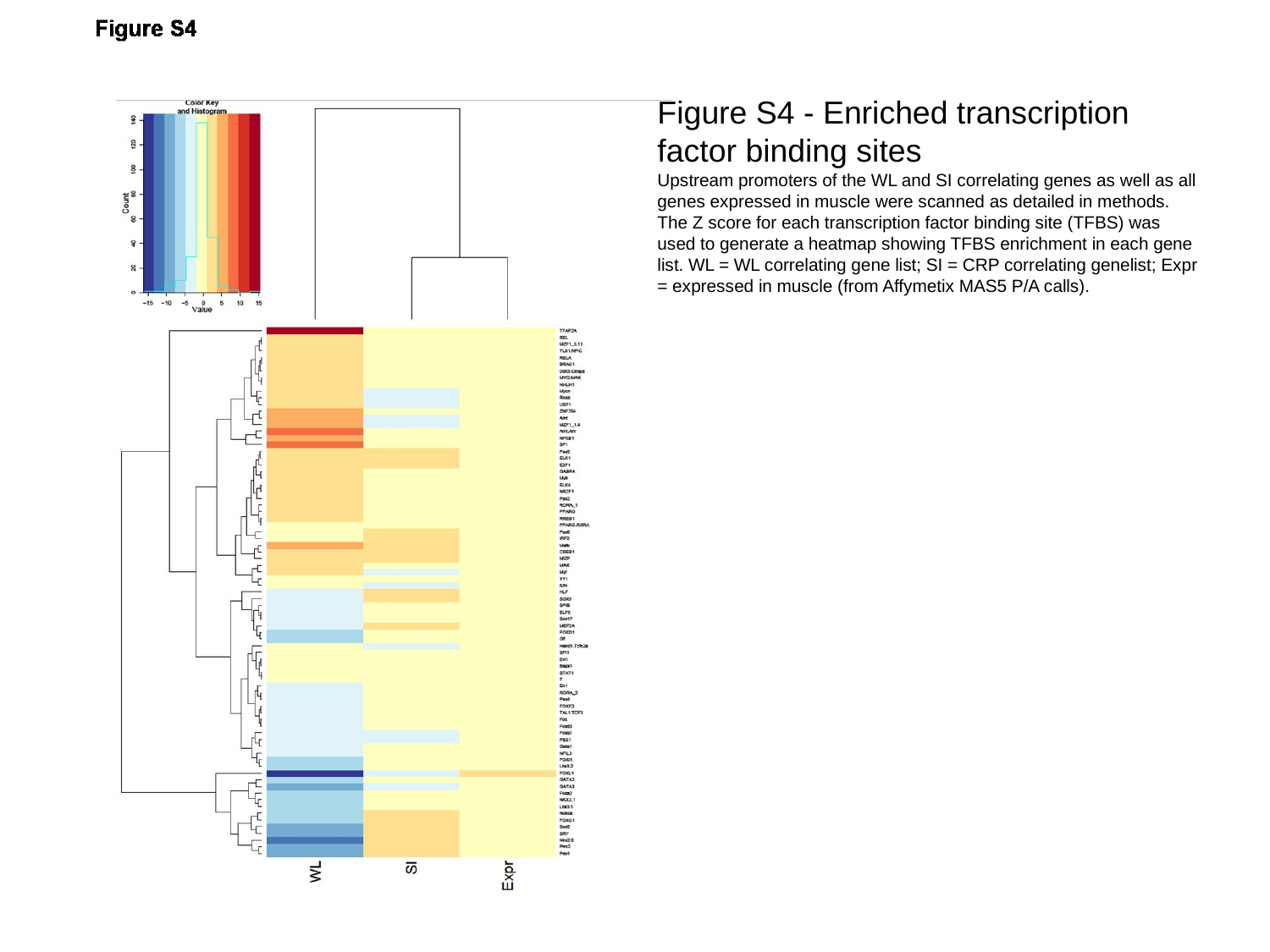

Figure S4 - Enriched transcription factor binding sites
Upstream promoters of the WL and SI correlating genes as well as all genes expressed in muscle were scanned as detailed in methods. The Z score for each transcription factor binding site (TFBS) was used to generate a heatmap showing TFBS enrichment in each gene list. WL = WL correlating gene list; SI = CRP correlating genelist; Expr = expressed in muscle (from Affymetix MAS5 P/A calls).
